# A Comprehensive Review of the Current Monkeypox Outbreak

**DOI:** 10.7759/cureus.34807

**Published:** 2023-02-09

**Authors:** Abdallah Kamal, Mustafa Suppah, Rakan Saadoun, Mohamed Yassin

**Affiliations:** 1 Oncology, University of Pittsburgh Medical Center, Pittsburgh, USA; 2 Internal Medicine, Mayo Clinic, Phoenix, USA; 3 Otolaryngology, Ruprecht Karls University Heidelberg, Mannheim, DEU; 4 Infectious Diseases, University of Pittsburgh Medical Center, Pittsburgh, USA

**Keywords:** monkeypox prevention, monkeypox transmission, monkeypox virus rash, monkeypox diagnosis, monkeypox outbreak, infection microbiology, clinical virology, public health and safety, monkeypox virus

## Abstract

*Monkeypox* is a zoonotic disease caused by an orthopoxvirus named monkeypox virus. The virus was identified in 1958, while the first human monkeypox case was discovered in 1970. Monkeypox caused a wide outbreak that was considered a global health emergency in July 2022. Monkeypox is transmitted through direct or indirect contact with the lesions and respiratory droplets. Animals can also transmit the disease if contacted without protection or if their products are consumed without proper processing. The disease presents as a prodromal period followed by the appearance of a rash filled with exudate. The rash appears initially on the face and then spreads to involve the genital area and the anus. Typically, the disease is mild and resolves spontaneously, but antiviral therapy with tecovirimat might be required. Monkeypox can be controlled by avoiding contact with the cases and vaccinating those at high risk for acquiring the infection and those at high risk for developing severe illness (immune deficient individuals, pregnant women, and children). Our review aims to comprehensively review the current literature regarding Monkeypox, including modes of transmission, pathogenesis, clinical presentation, diagnosis, treatment, preventive measures, and epidemiology.

## Introduction and background

Monkeypox is a zoonotic disease caused by the monkeypox virus, an enveloped ds-DNA virus that belongs to the Poxviridae family, Orthopox genus. Monkeypox virus shares the same genus with many other viral species; among those, only four viruses infect humans; monkeypox, vaccinia, cowpox, and variola viruses [1]. Developing immunity against each of these viruses can subsequently cross-protect against the others. The Monkeypox virus has 96% genomic sequence similarity with the variola virus that causes smallpox [2]. Genetic studies suggest that the two viruses originated independently from other poxviruses that infect rodents [3,4]. Another similarity was noticed between the monkeypox virus and the vaccinia virus used to produce smallpox vaccines [5]. 

Many strains of the monkeypox virus are present and are divided into clades. The first clade is the Central African or Congo Basin clade, which is more transmissible and pathogenic, resulting in more severe disease with an 11% case fatality rate (CFR). The first documented case of Monkeypox was caused by a monkeypox virus of the Central African clade [6]. This clade was identified in the Democratic Republic of Congo (DRC), Cameroon, Sudan, Gabon, and the Central African Republic. Moreover, this clade is more prevalent and endemic in the DRC [7-10]. The second clade is the West African clade, which is less pathogenic, with a lower-case fatality rate. This clade has been identified in Nigeria, Sierra Leone, Ivory Coast, and Liberia. In addition, the West African clade is responsible for the identified monkeypox cases outside Africa (US, UK, Singapore, and Israel) [11-13]. The current monkeypox outbreak is believed to be caused by a monkeypox virus of the West African clade [11,14,15]. However, it was also suggested that the current monkeypox outbreak is caused by a virus of a new clade [16].

The first identification of the monkeypox virus was in 1958 in Denmark at a research facility, as a pox-like disease outbreak occurred among macaque monkeys in the facility. Therefore, the disease and virus were named after the monkeys [17]. In 1970, human Monkeypox was reported in a baby aged nine months in the DRC [6]. The current outbreak's first reported case was an adult that traveled from Nigeria to the UK, and the virus was identified on May 6, 2022 [18]. Since the announcement of smallpox eradication in 1980, vaccination programs against smallpox have been terminated [19]. Subsequently, the cross-immunity against other orthopoxviruses has decreased over time. Therefore, changes in monkeypox virus virulence might worsen the disease outcome. By June 22, 2022, a total of 3,417 cases of Monkeypox were identified and confirmed across 58 countries on different continents. As a result, the World Health Network (WHN) declared Monkeypox a pandemic [20].

This review article summarizes the literature findings on monkeypox transmission, pathogenicity, case presentation, diagnosis, treatment, and preventive measures. In addition, the epidemiology of the disease will be described.

## Review

Transmission of the virus

Monkeypox is a zoonotic disease that can spread from animal to human as well as from human to human. In addition, the condition can have an asymptomatic circulation among humans [21,22]. A transmission of Monkeypox from forest animals (including monkeys and rodents) to humans was reported [23]. This animal-to-human transmission was suggested to occur following direct contact with the body fluids and skin or mucosal lesions of the infected animals or their bite. Moreover, consuming inadequately processed meat or other products of these animals can put a risk of acquiring the infection [24].

Different routes for human-to-human monkeypox dissemination were reported, among which close physical contact is the primary method [25]. Monkeypox virus can be acquired by contacting the patient's skin/mucosal lesions, infected body fluids, or respiratory droplets [26-28]. Currently, the majority of the cases were young men who had recent sexual contact with another new man or multiple men. Therefore, it was suggested that close physical contact during sexual activities is the primary mode of transmission in the 2022 outbreak [26]. On the other hand, indirect transmission can occur by contacting an object (e.g., clothing, towels, dishes) that is contaminated with the infective material [29].

Moreover, vertical transmission can occur across the placenta during pregnancy, resulting in a congenital monkeypox infection and sometimes fetal death [30,31]. No transmission through blood transfusion cases has been reported; this might be attributable to the strictly followed blood screening programs [24]. The primary source of infection in the current outbreak is still unknown [32].

Pathogenesis of the disease

Among orthopoxviruses that infect humans, the monkeypox virus is the second most virulent species after the smallpox virus [1]. In addition, several genetic mutations were identified in the monkeypox virus, causing the current outbreak, indicating a possibility of genomic alteration to adapt to the human body [17].

Once the virus is inoculated into the body, it starts replication locally. After that, the virus is carried out to circulate through the lymphatic system causing primary viremia after 10 - 14 days. After that, the virus replicates again in different lymphoid tissues and infects the epithelium and other organs. Many studies have suggested that the monkeypox virus can infect most mammalian cells [33,34]. Monkeypox virus - like other poxviruses - has several mechanisms to evade the host's immune defenses (from recognition to being targeted) [35].

Clinical presentation

Following infection with the monkeypox virus, the disease has an incubation period that ranges from three days to three weeks [15,36]. After that, the symptomatic phase may take another three to four weeks [24,36]. The clinical presentation of the disease begins with a four- to five-day prodromal period. In this prodromal period, the patients may experience fever with or without chills, headache, myalgia and lethargy, sore throat and cough, and lymphadenopathy [37]. In contrast to monkeypox cases in the epidemic areas, cases of the current outbreak experience prodromal symptoms less commonly (as 42% of patients did not experience prodromal symptoms) [28,38].

Monkeypox rash begins in small macules with a diameter of 2-5 mm. After that, the macules develop into papules, vesicles, and pustules [35,39-41]. Monkeypox skin/mucosal lesions are painful, circumscribed, filled with a yellowish or clear fluid, and possess central depression. The lesions heal by the first or second week as they become itch, dry, and fall off [42]. Typically, monkeypox rash appears on the face, spreads through the body, and involves the palms and soles. The lesion might be few, and there might also be thousands. However, the pattern of rash in the current outbreak is slightly unusual. Several studies have reported the involvement of the genital area, anus, oral mucosa, and eyes. Anal pain and bleeding were also reported, as well as having Monkeypox without any single lesion [28,35,36,38,43-45].

Typically, Monkeypox is a mild disease that resolves without requiring medical attention within two to four weeks [36,46]. However, the disease tends to be more severe in pregnant women, children, and immunocompromised patients [31,47,48]. Several conditions, including encephalitis, bronchopneumonia, myocarditis, acute renal injury, and sepsis, can complicate Monkeypox. In addition, involvement of the eyes (keratitis) can lead to impairment of vision over the long term [7,36,46]. Overall, monkeypox CFR during this outbreak is 0.03% among adults and 19% among children (<16 years) [49]. However, more deaths occur in Africa; this might be attributed to the lack of healthcare services required to treat complications and severe cases [50].

Diagnosis of Monkeypox

By January 1, 2022, the World Health Organization (WHO) defined the criteria for suspected monkeypox cases. The criteria included any person who presents with an acute skin lesion or rash and a prodromal symptom of Monkeypox. If another cause could not explain these presenting symptoms, a person is considered a suspected monkeypox case [51]. In addition, all suspected monkeypox cases, contacts, and patients with sexually transmitted disease that is not responsive to empiric therapy should be eligible for laboratory testing for the monkeypox virus. The diagnosis of Monkeypox could be confirmed by testing for monkeypox virus-specific DNA using the polymerase chain reaction (PCR) assay. The specimen could be taken via a swab from any lesion's exudate, surface, or crust [24,36]. The Serological test is an alternative if PCR is not available. The elevated IgM level can be detected after five days of the onset of the symptom, while elevated IgG levels can be detected after eight days [52,53]. Serological tests come positive when the lesions are already there [46]. When observed under an electron microscope, cells infected with the monkeypox virus exhibit a brick-like appearance [54].

Treatment of Monkeypox

Generally, Monkeypox is a mild disease that might only require supportive therapy [55]. However, some patients risk developing severe disease that requires hospitalization and treatment. Treatment with antiviral drugs is recommended for those having a high risk and those who have already developed a severe illness [56]. No specific antiviral drugs were developed for Monkeypox. However, the antiviral agents used for smallpox have shown efficacy in treating monkeypox [56,57].

Tecovirimat is the current treatment of choice for Monkeypox among adults and children (>3 Kg) [58]. This drug acts by inhibiting the localization of viral envelop intracellularly, thus interfering with viral maturation and release [59]. Tecovirimat can be administered orally or intravenously; it has shown efficacy in treating Monkeypox with minimal side effects and safety during pregnancy [46,57]. Cidofovir and brincidofovir are other antiviral agents used for treating Monkeypox. Brincidofovir is an oral prodrug of cidofovir, while cidofovir can be administered topically and intravenously. These drugs act by inhibiting the viral DNA polymerase, thus interfering with viral replication [56]. Although cidofovir and brincidofovir have shown efficacy in treating Monkeypox, they are less effective than tecovirimat and have caused teratogenicity and elevated liver enzymes [46]. 

Vaccinia immunoglobulin -originally developed to treat the complications of the vaccinia vaccine - has been used intravenously to treat Monkeypox. This immunoglobulin has shown efficacy in treating Monkeypox and safety (especially during pregnancy) [36].

Preventive measures

After the smallpox vaccination programs were terminated following the disease eradication, the immunity against orthopoxviruses has been waning. In addition, the monkeypox virus has animal reservoirs. All these factors make the control of monkeypox dissemination a significant challenge. However, several preventive methods can be applied to limit disease dissemination. As Monkeypox is a zoonotic disease, preventive methods should be considered when dealing with animals, especially in endemic regions. Contact with wild animals, especially the sick or dead ones, should be protected. In addition, animal products should be adequately cooked [24].

Awareness programs should be carried out to educate the public about the symptoms of Monkeypox. Knowing the characteristic symptoms of the disease will enable the individuals to suspect Monkeypox and subsequently reach out to any health facility for early diagnosis [60,61]. In addition, minimizing the stigma associated with monkeypox transmission would reduce individuals' hesitancy to seek medical care [62]. Broad access to rapid diagnostic tests and antiviral therapy is required, as early diagnosis and treatment of the cases would limit the dissemination of the virus [63]. The identified cases of Monkeypox should be isolated (to limit respiratory transmission), and the lesions must be kept covered with proper disposal of the fomites (to limit contact transmission) [26,27,29].

Moreover, individuals are advised to reduce the number of their sexual partners to reduce the possibility of acquiring Monkeypox. In addition, medical workers are encouraged to wear face shields, respirator masks, long-sleeved gowns, and disposable gloves when dealing with a suspected monkeypox case [24,64]. Identifying and vaccinating people in contact with monkeypox patients minimizes the incidence of secondary cases by 86.1% [65]. Hands should be adequately washed using soap or alcohol, and any contaminated surface should be cleaned [66].

The smallpox vaccine has shown cross-protectiveness against Monkeypox, as the monkeypox virus is antigenically similar to the vaccinia virus [67]. Therefore, individuals at high risk of contracting the monkeypox virus (such as health workers, scientists, and gay or bisexual men with multiple partners) are encouraged to have the vaccine [64, 68-71]. In addition, individuals at risk of developing severe illness (such as immune-deficient individuals, children, and pregnant women) are advised to take the vaccine before exposure [58, 72]. Studies on the smallpox vaccine have revealed that it has offered 85% protection against Monkeypox and has reduced its attack rate (7.2% for unvaccinated contacts versus 0.9% for the vaccinated ones) [73,74]. Furthermore, the vaccine has limited monkeypox mortality (11% versus 0%), and the vaccinated individuals have remained immune even after 25 years [7,75]. Because of the prolonged incubation period, the vaccine can be administered up to days after exposure to the virus [76].

Currently, two smallpox vaccines (previously approved by the food and drug administration) are used for Monkeypox; the modified vaccinia Ankara and ACAM2000 vaccine. The modified vaccinia Ankara is a vaccine made of a non-replicating live attenuated vaccinia virus. The vaccine is administered in two subcutaneous doses separated by one month. The modified vaccinia Ankara offers protection with a good safety profile, as it can be used in immune-deficient individuals, pregnant women, and patients with skin disorders. Minimal side effects were reported, including pain at the injection site, headache, muscle aches, and lymphadenopathy. However, these side effects lasted only a few days after receiving the vaccine [69,77]. The ACAM2000 vaccine is made of a vaccinia virus capable of replication, thus, should not be administered to immune-deficient individuals or pregnant women. This vaccine is administered as a single dose using a bifurcated needle into the epidermis (known as the multi-puncture technique). The experience side effects range from mild effects that last for one to two weeks (such as local erythema and pain, fever and chills, muscle aches and fatigue, nausea, and headache) to severe side effects (such as encephalitis, myocarditis, and pericarditis) [69,78].

Epidemiology of Monkeypox

After being isolated from monkeys in 1959, the monkeypox virus has caused several outbreaks among different animal species [79]. In 1970, the first occurrence of human Monkeypox was reported in a 9-months baby from a small remote village in the DRG [1]. Since then, several cases have been reported in Africa, and Monkeypox has become endemic in Central and West Africa [23,25]. However, the majority of cases were from the DRC [17]. The year 2003 witnessed the first monkeypox outbreak outside of Africa, as a limited outbreak in the US was caused by an imported pet from Ghana [80]. After that, other outbreaks occurred, and they were all linked to a travel history from the endemic regions of Africa. These include the 2018-19 outbreak in the UK, the 2018 outbreak in Israel, the 2019 outbreak in Singapore, and the 2021 outbreak in the US [81].

The first reported case in the current outbreak was of a male who traveled from Nigeria to the UK in May 2022. However, this patient has denied a history of contact with an infected person; therefore, the source of this outbreak is still unknown [82]. Within the following months, cases of Monkeypox have been increasing in number and spread through more than 50 countries over six continents [72]. By July 23, 2022, Monkeypox was declared by the WHO a global health emergency [83]. As the immunity against poxviruses has waned by terminating smallpox vaccines and as they travel between different countries has become more accessible, Monkeypox has disseminated globally [31]. A remarkable accumulation of the disease was observed among men who have sex with men and the minor ethnic groups [38]. Monkeypox cases were more prevalent in the DRC, Spain, the UK, Germany, Portugal, and France [72].

Cases presentation

Twenty-two patients were admitted to our medical center and diagnosed with Monkeypox using real-time PCR. Of them, twenty patients were adults with a mean age of 34.45 years (standard deviation = 11.65), while only two patients were neonates with an age of 20 days. Most adult patients were admitted and released on the same day or after one day, with only one old patient staying three days, while for neonates, the hospital stay was 14 days. The complaints at diagnosis for adult patients were varying as follows: flu symptoms, sore throat, rash, vomiting, lesion on stomach and groin, painful groin, rectal pain, penile pain, and rectal bleeding, with the flu-like symptoms and rash being the most frequent adults' complaints, while the neonates had sepsis.

## Conclusions

The current monkeypox outbreak is of global concern, as declared by the WHO. Efforts are required to diagnose the cases early, treat them properly, and control further virus dissemination. Monkeypox cases should be isolated, and their lesions should be kept closed. In addition, vaccination of the contacts, scientists, and medical workers is required. Other individuals at high risk of acquiring the infection (bisexual men and gays) are also encouraged to take the vaccine. In addition, individuals having an increased risk of developing severe disease (immunocompromised individuals, pregnant women, and children) are also encouraged to take the non-replicating vaccine.
